# A Multicenter Retrospective Study of Avelumab First-Line Maintenance and Subsequent Therapies for Locally Advanced and Metastatic Urothelial Carcinoma: Subgroup Analysis of First-Line Dose-Dense Methotrexate, Vinblastine, Doxorubicin, and Cisplatin, and Gemcitabine Plus Cisplatin in the Japan AVElumab MAintenance and Continuous Treatment Study (JAVEMACS)

**DOI:** 10.3390/curroncol32110618

**Published:** 2025-11-05

**Authors:** Masaomi Ikeda, Kiyohide Fujimoto, Noriyoshi Miura, Rikiya Taoka, Kiyoaki Nishihara, Daiki Ikarashi, Sei Naito, Fumitaka Shimizu, Atsuko Fujihara, Michihiro Shono, Tohru Nakagawa, Eiji Kikuchi

**Affiliations:** 1 Department of Urology, School of Medicine, Kitasato University, Sagamihara 252-0374, Japan; 2 Department of Urology, Nara Medical University, Kashihara 634-8521, Japan; kiyokun@naramed-u.ac.jp; 3 Department of Urology, Graduate School of Medicine, Ehime University, Toon 791-0295, Japan; norimiurajp@yahoo.co.jp; 4 Department of Urology, Faculty of Medicine, Kagawa University, Takamatsu 760-8521, Japan; taoka.rikiya@kagawa-u.ac.jp; 5 Department of Urology, School of Medicine, Kurume University, Kurume 830-0011, Japan; nishihara_kiyoaki@kurume-u.ac.jp; 6 Department of Urology, School of Medicine, Iwate Medical University, Yahaba 028-3694, Japan; dikara@iwate-med.ac.jp; 7 Department of Urology, Faculty of Medicine, Yamagata University, Yamagata 990-9585, Japan; seinaitoh@yahoo.co.jp; 8 Department of Urology, Graduate School of Medicine, Juntendo University, Tokyo 113-8421, Japan; f_simizu@juntendo.ac.jp; 9 Department of Urology, Kyoto Prefectural University of Medicine, Kyoto 602-8566, Japan; fujihara@koto.kpu-m.ac.jp; 10 Medical Department, Merck Biopharma Co., Ltd. (An Affiliate of Merck KGaA, Darmstadt, Germany), Tokyo 106-0041, Japan; michihiro.shono@merckgroup.com; 11 Department of Urology, School of Medicine, Teikyo University, Tokyo 173-8605, Japan; nakagawat@med.teikyo-u.ac.jp; 12 Department of Urology, School of Medicine, St. Marianna University, Kawasaki 216-8511, Japan; eiji-k@kb3.so-net.ne.jp

**Keywords:** treatment patterns, ddMVAC, GC, avelumab, enfortumab vedotin, urothelial carcinoma

## Abstract

**Simple Summary:**

Avelumab maintenance therapy is approved in Japan for patients with advanced urothelial carcinoma (aUC) who have not seen their cancer progress after receiving platinum-based chemotherapy (PBC). This study provides important information about how effective avelumab is for these patients. It shows that survival rates for patients who received either dose-dense methotrexate, vinblastine, doxorubicin, and cisplatin (ddMVAC) or gemcitabine plus cisplatin (GC) are similar to those in the overall group, even though there are differences among the subgroups. The findings emphasize the need to choose the best first-line (1L) PBC and to start avelumab treatment promptly. The study suggests that using ddMVAC or GC followed by avelumab and then enfortumab vedotin (EV) can lead to better long-term health outcomes for patients.

**Abstract:**

Avelumab maintenance therapy is approved in Japan for patients with aUC without progression after PBC. This report presents subgroup analysis data from the JAVEMACS chart review of avelumab maintenance in patients who received 1L ddMVAC and GC. This retrospective study reviewed medical charts of patients with aUC (February 2021–December 2023). Overall, 350 patients (ddMVAC, *n* = 32 and GC, *n* = 196) were included in the study. Baseline characteristics were balanced between the two PBC groups. Median duration from PBC start to avelumab start was 13.2 and 21.1 weeks; median overall survival (OS) was not reached (both groups), progression-free survival (PFS) was 12.0 and 7.4 months, and PFS2 was 27.6 and 21.3 months for the ddMVAC and GC groups, respectively. At data cutoff (June 2024), 25.0% of patients in the ddMVAC and 17.3% in the GC groups were ongoing avelumab treatment. Second-line treatments included EV (64.3% ddMVAC; 64.5% GC), pembrolizumab (21.4% ddMVAC; 8.3% GC), and PBC (14.3% ddMVAC and 21.5% GC). This real-world data from patients with aUC in Japan showed consistent OS patterns with avelumab maintenance across treatment subgroups vs. the overall population despite their inherent heterogeneity. Although patients were not resistant to PBC, 2L EV was more common than 2L PBC.

## 1. Introduction

Urothelial carcinoma (UC) is a cancer of the urothelium encompassing the renal pelvis, ureter, bladder, and urethra [1] with over 90% of UC cases originating in the bladder [2]. Globally, UC accounted for 614,298 new cancer cases and over 220,596 deaths in 2022 [3]. In Japan, approximately 24,800 new UC cases and 9900 deaths due to bladder cancer were reported in 2023 [4].

For over two decades, cisplatin plus gemcitabine (GC) has been the standard first-line (1L) treatment approved for patients with locally advanced or metastatic UC (la/m UC) and is recommended by guidelines worldwide, including Japan [2,5,6,7,8]. In patients with aUC, treatment with platinum-based chemotherapy (PBC) especially with GC, achieved an objective response rate (ORR) of over 40% and a disease control rate (DCR) of approximately 75%; however, the median overall survival (OS) was only about 12 to 16 months [9,10,11,12,13,14].

A Phase 3 study has demonstrated that while GC is the preferred regimen because of its better tolerability, methotrexate, vinblastine, doxorubicin, and cisplatin (MVAC) and GC have comparable efficacy in treating patients with metastatic bladder cancer [13]. The EORTC Phase 3 study comparing dose-dense (dd)MVAC and conventional MVAC in aUC revealed that ddMVAC was better tolerated than conventional MVAC and showed a trend towards longer progression-free survival (PFS) and OS rates, with a median OS of 15.1 months [15]. Although ddMVAC has not been extensively confirmatory in aUC, the VESPER trial, which compared ddMVAC to GC in the perioperative setting, suggested that ddMVAC provides similar or improved response rates and survival compared to GC, with a favorable tolerability profile, when used as neoadjuvant therapy [16,17].

The treatment landscape for la/m UC is rapidly evolving, driven by the need to improve survival outcomes. Recent advancements include the approval of various immune checkpoint inhibitors (avelumab, nivolumab, pembrolizumab), antibody-drug conjugates (enfortumab vedotin [EV]) and fibroblast growth factor receptor inhibitors (erdafitinib) [2,7].

Avelumab, an anti–PD-L1 monoclonal antibody, was approved in many countries as a maintenance treatment for patients with la/m UC without disease progression on PBC [2,5,7,8,18]. The approval was based on the results of the Phase 3 JAVELIN Bladder 100 which demonstrated a significant improvement in median OS with avelumab plus best supportive care (BSC) as 1L maintenance versus (vs) BSC alone (median OS, 21.4 months vs. 14.3 months), resulting in a 31% reduction in the death risk in the overall population (hazard ratio [HR] = 0.69 (95% confidence interval [CI]: 0.56–0.86), *p* = 0.001) [19]. Long-term follow-up from this trial also confirmed efficacy and safety of avelumab 1L maintenance in patients with aUC [20]. Subgroup analysis in Japanese patients led to its approval in Japan in 2021 [21]. However, to date, there is a dearth of long-term studies evaluating the treatment patterns of avelumab maintenance from 1L to subsequent lines following 1L ddMVAC in patients with la/m UC in daily clinical practice. Therefore, this subgroup analysis of the Japan AVElumab Maintenance And Continuous treatment Study (JAVEMACS) aims to evaluate the baseline characteristics, treatment patterns, and effectiveness in patients with la/m UC who received avelumab maintenance following 1L PBC with 1L ddMVAC and compare it alongside GC group.

## 2. Materials and Methods

### 2.1. Patients

JAVEMACS was a multicenter, non-interventional, retrospective, medical chart review of patients with la/m UC conducted across 26 sites in Japan including university hospitals and cancer institutes. Patients who received 1L ddMVAC included in this analysis were recruited from 11 participating institutions; all 11 institutions also registered 1L GC patients. Regimen selection (ddMVAC or GC) was at the discretion of the treating physicians in each facility under routine clinical practice, and local treatment policies may have evolved during the enrollment window.

Participants included those who did not have disease progression following 1L PBC and who have started avelumab 1L maintenance therapy between 24 February 2021 (date of regulatory approval in Japan) and 6 months before the date of approval of implementation of the study at each site. The study involved urologists with expertise in the treatment of la/m UC. Participants’ data were retrospectively collected when available, at each study site, using an electronic case report form. Data collection spanned from the start date of avelumab maintenance therapy until loss to follow-up, any-cause death, or the end of data collection period, whichever occurred first.

This study was conducted in accordance with the Declaration of Helsinki and the “Ethical Guidelines for Life Science and Medical Research Involving Human Subjects” issued in Japan. Study protocol, amendments, and other relevant documents were prospectively approved by Non-Profit Organization MINS Research Ethics Committee (Approval No. MINS-REC-240211; approval date: 4 April 2024) and posted on ClinicalTrial.gov (ID: NCT06412848; approval date: 10 May 2024).

### 2.2. Study Outcomes

Primary outcomes included baseline demographics and clinical characteristics of patients, characteristics of 1L PBC prior to avelumab maintenance and subsequent treatment patterns of avelumab maintenance during second-line (2L) or later lines. Secondary outcomes included OS (from start of avelumab maintenance therapy and from start of 1L PBC), PFS, PFS2 (time from the initial date of a therapy to the date of first disease progression after the next-line therapy or death, whichever occurs first), ORR and DCR.

### 2.3. Statistical Analysis

Continuous variables were summarized using descriptive statistics, while qualitative variables were summarized by frequency counts and percentages. The Kaplan–Meier method was used to estimate the time-to-event endpoints (OS, PFS). Univariate Cox regression analysis was conducted to calculate the HR and the 95% CI for OS and PFS. For ORR and DCR, the number and proportion of patients and the corresponding 95% CI were reported using the Clopper-Pearson method. It is important to note that no adjustments for confounding factors were made in this analysis, as the focus was primarily on examining the effects of individual factors on survival outcomes. Missing values were summarized using the number and proportion of missing values. No data imputation was performed. All analyses were performed using SAS^®^ Software version 9.4 or later.

## 3. Results

### 3.1. Patient Characteristics

Between February 2021 and December 2023, a total of 360 patients were screened across 26 centers, including university hospitals and cancer institutes in Japan. Each center enrolled eligible patients, and of these, 10 patients were found ineligible after registration in the electronic data capture system, resulting in 350 patients being analyzed. Patients who received 1L ddMVAC came from 11 centers, all of which also registered 1L GC patients. This mixed-regimen contribution across centers supports the subgroup comparisons but may introduce site-level heterogeneity given non-interventional regimen selection.

At the data cutoff (June 2024), the median observation period from avelumab initiation was 14.6 months (interquartile range [IQR]: 9.7–23.9), while the median duration of avelumab treatment was 14.3 weeks (95% CI: 7.1–30.9).

Baseline demographics and clinical characteristics in subgroups defined by 1L ddMVAC (*n* = 32) and 1L GC (*n* = 196) at avelumab initiation are shown in Table 1. At the time of avelumab initiation, the median age was comparable: 71.0 years vs. 72.0 years for the ddMVAC group vs. GC group, respectively. Notably, 3 (9.4%) patients in the ddMVAC group and 23 (11.7%) patients in the GC group were aged 80 years or older. There was no difference in Eastern Cooperative Oncology Group performance status (ECOG PS), creatinine clearance (CCr), or body mass index between the two groups (Table 1).

A notable trend was identified in laboratory results between the start of 1L PBC and start of avelumab maintenance therapy in both groups; the percentage of patients with hemoglobin levels <10 g/dL tended to rise at the start of avelumab maintenance therapy, whereas the percentage of patients with inflammatory markers such as C-reactive protein (CRP) levels <0.3 mg/dL tended to decline from the start of 1L PBC to start of avelumab maintenance therapy. Additionally, a higher proportion of patients with ECOG PS 1 was observed in the ddMVAC group, while no changes were noted in the GC group. CCr remained relatively stable in both groups. (Supplementary Table S1).

At the initiation of 1L PBC, 16 (50.0%) patients in the ddMVAC group and 78 (39.8%) patients in the GC group were eligible for cisplatin, while 14 (43.8%) patients in the ddMVAC group and 91 (46.4%) patients in the GC group were ineligible for cisplatin but eligible for PBC. This eligibility was determined retrospectively based on the cisplatin eligibility criteria of Galsky et al. [22] and platinum ineligibility criteria of Gupta et al. [23]. The median number of cycles for 1L PBC were 4, with 22 (68.8%) patients in the ddMVAC group and 109 (55.6%) patients in the GC group receiving 4 cycles. There were no patients in the ddMVAC group who received 7 cycles or more. The median duration between the initiation of 1L PBC and avelumab initiation was 13.2 weeks for the ddMVAC group and 21.1 weeks for the GC group. Cisplatin dose reductions in PBC were noted, with 14 (43.8%) patients in the ddMVAC group and 74 (37.8%) patients in the GC group experiencing reductions; of these, 8 (25.0%) and 37 (18.9%) patients had their doses reduced from the first cycle, while 3 (9.4%) and 20 (10.2%) patients had reductions from second cycle (Table 2).

The median treatment-free interval (TFI) between the end of 1L PBC and avelumab initiation for the two groups are shown in Table 2. The best overall response (BOR) rates indicated that 3 (9.4%) patients in the ddMVAC group and 21 (10.7%) patients in the GC group achieved a CR, while 21 (65.6%) and 103 (52.6%) achieved a partial response (PR), and 8 (25.0%) and 72 (36.7%) had stable disease (SD) (Table 2).

### 3.2. Subsequent Treatment

**Figure 1:**
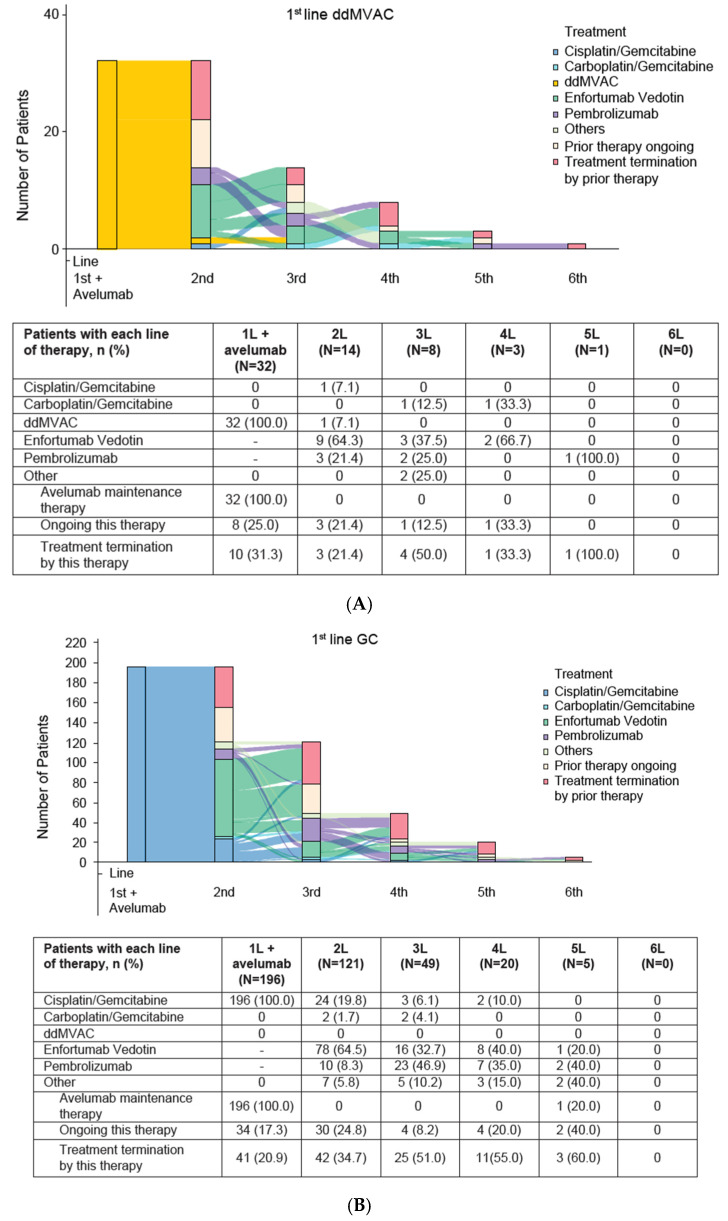
Treatment patterns in 1L (**A**) ddMVAC and (**B**) GC. ddMVAC, dose-dense methotrexate, vinblastine, doxorubicin, and cisplatin; GC, gemcitabine + cisplatin; L, line.

At the data cutoff, 8 (25.0%) patients in the ddMVAC group and 34 (17.3%) patients in the GC group were still receiving avelumab maintenance therapy. Following discontinuation of avelumab, 14 patients in the ddMVAC group and 121 in the GC group, i.e., 58.3% and 74.7% of discontinuing patients in each group received 2L treatment, respectively. Among these, 9 (64.3%) patients in the ddMVAC group and 78 (64.5%) patients in the GC group received EV, while 3 (21.4%) and 10 (8.3%) received pembrolizumab, and 2 (14.3%) and 26 (21.5%) received additional PBC. EV was used in 3 (37.5%) and 16 (32.7%) patients who proceeded to third-line (3L) treatment and 2 (66.7%) and 8 (40.0%) of patients who proceeded to fourth-line (4L) treatment (Figure 1A,B).

### 3.3. Effectiveness

**Figure 2:**
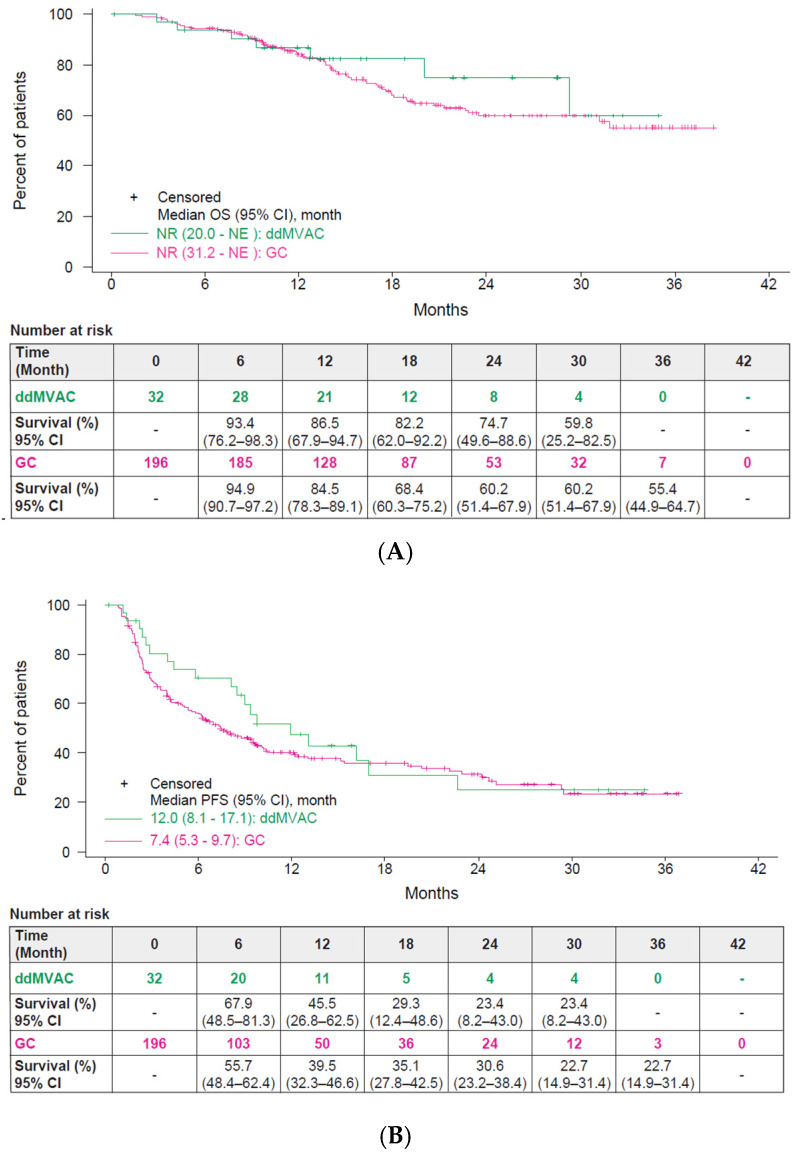

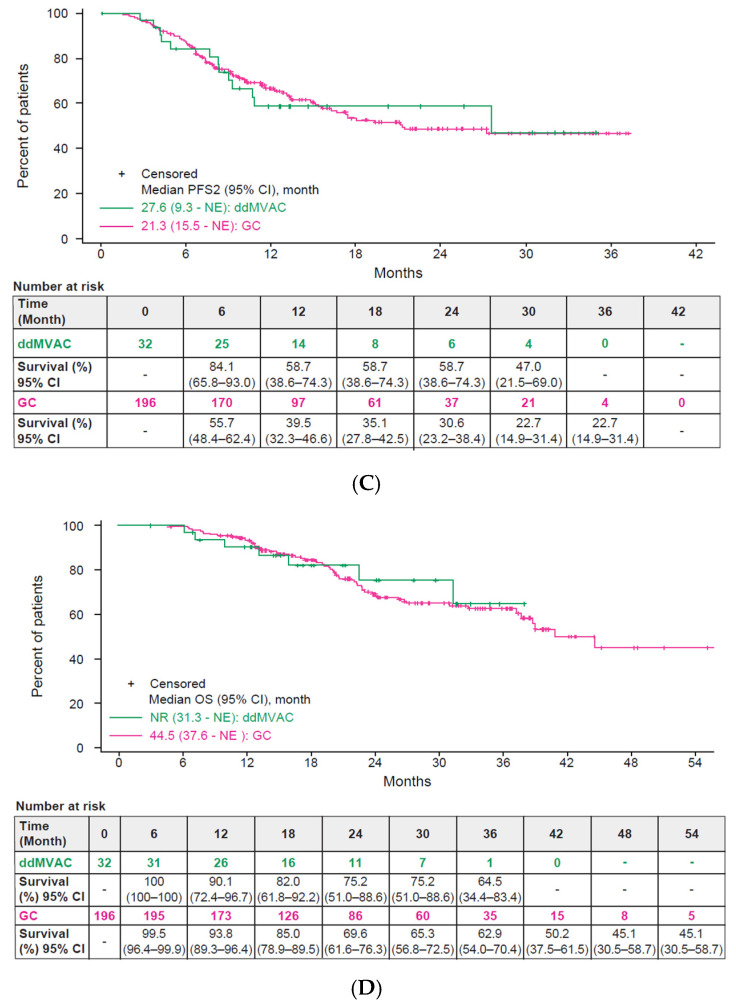
Kaplan–Meier curves for (**A**) OS from avelumab maintenance therapy; (**B**) PFS from avelumab maintenance therapy; (**C**) PFS2 from avelumab maintenance therapy; (**D**) OS from 1L PBC by subgroups on PBC regimen (ddMVAC, cisplatin/gemcitabine). CI, confidence interval; ddMVAC, dose-dense methotrexate, vinblastine, doxorubicin, and cisplatin; GC, gemcitabine + cisplatin; OS, overall survival; PBC, platinum-based chemotherapy; PFS, progression-free survival; PFS2, progression-free survival 2.

The median OS from the start of avelumab was not reached (NR) for the ddMVAC group (95% CI:20.0–NE; 7 events) and NR for the GC group (95% CI:31.2–NE; 61 events), with an HR of 0.77 (95% CI: 0.35–1.69). Landmark OS rates at 12 and 24 months were 86.5% and 74.7% for the ddMVAC group, and 84.5% and 60.2% for the GC group (Figure 2A).

The median PFS was 12.0 months (95% CI:8.1–17.1; 19 events) for the ddMVAC group and 7.4 months (95% CI:5.3–9.7; 126 events) for the GC group, with an HR of 0.79 (95% CI: 0.49–1.29). The median PFS2 for the ddMVAC group was 27.6 months (95% CI:9.3–NE) and for the GC group was 21.3 months (95% CI:15.5–NE), with an HR of 0.95 (95% CI: 0.52–1.74) (Figure 2B,C). In an exploratory analysis of patients without progression following 1L chemotherapy, the median OS from the start of chemotherapy was NR (95% CI:31.3–NE) for the ddMVAC group and 44.5 months (95% CI:37.6–NE) for the GC group (Figure 2D).

**Figure 3:**
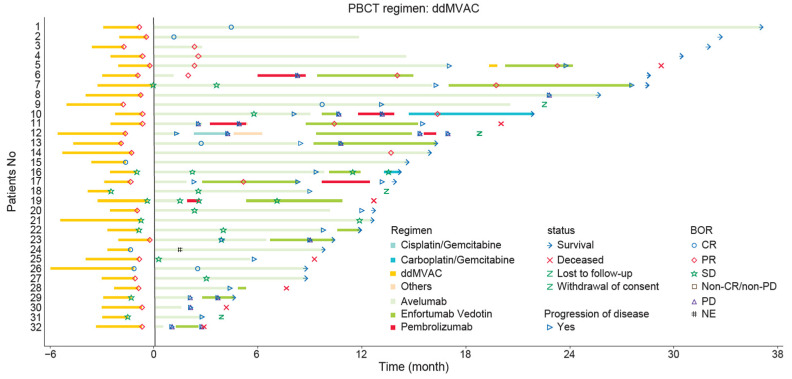
Swimmers plot for patients on 1L ddMVAC. BOR, best overall response; CR, complete response; ddMVAC, dose-dense methotrexate, vinblastine, doxorubicin, and cisplatin; GC, gemcitabine + cisplatin; NE, not evaluable; ORR, objective response rate; PBC, platinum-based chemotherapy; PR, partial response; PD, progressive disease; SD, stable disease.

The ORR during avelumab maintenance was 38.5% (95% CI:20.2–59.4) for the ddMVAC group and 24.9% (95% CI:18.6–32.0) for the GC group, while the DCR was 80.8% (95% CI:60.6–93.4) and 63.6% (95% CI:55.9–70.8), respectively (Table 3). Swimmer plots illustrating treatment responses in the subgroups of 1L ddMVAC in Figure 3.

## 4. Discussion

This subgroup analysis of JAVEMACS provides valuable insights into the real-world effectiveness of avelumab maintenance therapy following 1L ddMVAC, contributing to improved treatment strategies for patients with Ia/m UC. Our findings indicate a favorable OS and PFS for patients receiving avelumab, highlighting the potential of this treatment sequence in clinical practice.

Baseline characteristics tended to be largely comparable between the ddMVAC and GC groups, suggesting that the patient populations were well-matched for this analysis with exception of CCr, smoking history and bone metastasis. The median age of the patients and proportion of those >80 years were higher compared to the JAVELIN Bladder 100 [19]. Additionally, a higher proportion of patients had upper tract UC (UTUC) in those two groups, which is in contrast to findings from JAVELIN Bladder 100 [19] and other observational studies in the US and Europe [24,25,26]. The median CCr was numerically higher in the ddMVAC group (65.0 mL/min) compared to the GC group (57.2 mL/min). Despite undergoing 1L chemotherapy, regardless of whether it was GC or ddMVAC, there were no significant changes like weight loss (BMI) or deterioration in overall condition (ECOG PS) attributed to cisplatin. Although the proportion of patients with hemoglobin levels below 10 increased, which is expected due to the myelosuppression caused by cisplatin, inflammatory markers such as Neutrophil Lymphocyte Ratio and CRP showed improvement, and renal function, which was a major concern, was maintained. This indicates that the transition to avelumab maintenance therapy was conducted while effectively managing both tumor control and overall condition during 1L treatment. This may be a potential contributing factor to the favorable OS observed (Supplementary Table S1).

Around half of the patients in both ddMVAC and GC groups were platinum ineligible, there may be a need to consider alternative regimens such as gemcitabine plus carboplatin, but many patients were elderly, and primary tumors include multiple sites of UTUC. However, the survival benefits (OS and PFS) were the same or better than those of previous reports [19,26,27]. Median number of cycles for 1L PBC was four, with 68.8% of patients in the ddMVAC group receiving four cycles and only 18.8% of patients in the ddMVAC group receiving 6 cycles. In contrast to this, the VESPER trial [16,28] recommends six cycles of ddMVAC and transitioning to avelumab after four cycles may enhance tolerability.

An operational advantage of ddMVAC in our cohort was a shorter interval from PBC initiation to avelumab maintenance (median 13.2 weeks with ddMVAC vs. 21.1 weeks with GC), allowing for a limited intensive treatment period that may reduce patient burden. The swimmer plot reveals that patients who continued avelumab maintenance therapy and those who discontinued it prematurely without receiving 2L therapy were more frequently found among those with long survival periods. Completing ddMVAC within four 2-week cycles typically confines intensive chemotherapy to about two months and enables timely transition to avelumab maintenance; in our cohort, long-term survival was observed even after avelumab continuation or cessation, although these observations are hypothesis-generating.

The dose reductions were observed in 43.8% of the patients in ddMVAC group and 37.8% of the patients in the GC group. These factors highlight the importance of careful dose management in 1L therapies. The median TFI was 4.2 weeks for the ddMVAC group and 5.1 weeks for the GC group. In clinical practice, TFI is adjusted based on individual circumstances, as evidenced by the 37.5% of patients in the ddMVAC group initiating avelumab within four weeks, highlighting the need for flexible treatment strategies.

At data cutoff, 25.0% of patients in the ddMVAC group and 17.3% of patients in the GC group were still receiving avelumab maintenance therapy. Following discontinuation, 58.3% of ddMVAC and 74.7% of GC patients received 2L treatment, predominantly EV (64.3% and 64.5%, respectively). EV was used in a majority of patients in 3L and 4L, specifically in those patients who received 2L treatment. These rates surpass those observed in the JAVELIN Bladder 100 study [20], indicating a promising trend toward effective treatment sequencing. The high utilization of EV in 2L therapy after avelumab maintenance correlates with improved survival, as seen in the AVENANCE [26] and JAVEMACS populations, reinforcing the potential of avelumab maintenance therapy in 1L for enhancing outcomes for patients with aUC.

The median OS from the start of avelumab was NR for both the ddMVAC and GC groups; however, landmark OS rates at 12 and 24 months were promising, at 86.5% and 74.7% for ddMVAC, and 84.5% and 60.2% for GC, respectively. PFS appears to be longer than in the JAVELIN Bladder 100 trial (5.5 months) [20]. While PFS2 of avelumab maintenance therapy cannot be directly compared due to lack of prior reports, it appears favorable in this study. The strong OS results may be attributed to the study being conducted at a center with expertise in UC, potentially limiting generalizability across Japan. The favorable PFS and PFS2 may have contributed to OS, plausibly reflecting careful selection of 1L chemotherapy (regimen, number of cycles, dose adjustments) and timely transition to avelumab maintenance; however, given the retrospective design and potential site-selection effects, this interpretation should be considered exploratory. Additionally, the use of cisplatin, even with dose adjustments, in patients considered to be ineligible may have positively influenced OS outcomes, aligning with findings from the JAVELIN Bladder 100 and other studies [19,20,21,24,25,29]. The ORRs and DCRs were higher than those from the JAVELIN Bladder 100 [20] and previously reported observational studies [30,31], indicating that improved ORR and DCR, along with favorable PFS and PFS2, may enhance OS outcomes. Favorable ORR and DCR for avelumab maintenance therapy were observed in the 1L ddMVAC group compared to the 1L GC group. This may suggest that the enhanced immunogenicity obtained from each of the chemotherapy agents constituting ddMVAC was effectively translated to avelumab therapy in a relatively short time, resulting in a higher tumor reduction and maintenance effect from avelumab [32,33,34,35]. As this dataset comprises only patients who initiated avelumab maintenance after 1L PBC, regimen-specific transition rates to maintenance cannot be calculated from the full 1L PBC denominator, introducing potential selection bias. Prior to maintenance, the best overall response to 1L PBC differed between ddMVAC and GC (CR/PR/SD: 9.4%/65.6%/25.0% vs. 10.7%/52.6%/36.7%), and during maintenance, ORR and DCR were higher with ddMVAC (ORR 38.5% vs. 24.9%; DCR 80.8% vs. 63.6%). Although median PFS and PFS2 favored ddMVAC (12.0 vs. 7.4 months; 27.6 vs. 21.3 months), the OS curves were broadly similar, and the OS hazard ratio had wide confidence intervals (HR 0.77; 95% CI 0.35–1.69). Given the limited sample size in the ddMVAC subgroup (*n* = 32) and the retrospective, unadjusted analyses, these results should be considered hypothesis-generating rather than demonstrative of regimen superiority. Future large prospective studies with appropriate adjustment for confounding are needed to verify and quantify any comparative effectiveness signals.

Despite these promising results, it is essential to consider the limitations inherent in the study. Firstly, its retrospective design, which depended on existing medical record data for data collection and may have missing variables, could affect the accuracy of the estimations made. In this study, the univariate cox regression analysis was not adjusted for confounding factors, which may have introduced bias in the estimation of HR. The lack of adjustment for confounders limits the ability to draw definitive conclusions regarding the causal relationships between the factors analyzed and the observed outcomes. Additionally, the comparison of outcomes between imbalanced cohorts may not accurately reflect the true effect of the variables studied. Thus, while the unadjusted HRs provide preliminary insights, caution should be taken when interpreting these results, and future studies should include multivariate analyses to better control the potential confounding. Secondly, the preferential selection of centers of excellence for this study introduces the possibility of site selection and outcome reporting bias, which may not accurately represent clinical outcomes across all Japanese healthcare settings. The evaluation of disease response may vary between sites, leading to potential measurement errors in the reported values. Lastly, patients who received 1L ddMVAC came from 11 of the 26 participating institutions, and all 11 institutions also registered GC cases. Regimen selection was non-interventional and at physician discretion, and local policies may have evolved during enrollment. These factors may introduce site-level and temporal heterogeneity and should be considered when interpreting subgroup comparisons.

## 5. Conclusions

In this subgroup analyses, OS in the 1L ddMVAC and GC subgroups aligns with the overall population, despite the inherent subgroup heterogeneity, suggesting that treatment benefits are broadly applicable across different patient profiles. Choosing appropriate 1L PBC and the timely transition to avelumab maintenance therapy are crucial for enhancing long-term OS, regardless of the cisplatin eligibility. The proposed treatment sequence, which involves administering 1L ddMVAC or GC without disease progression, followed by avelumab maintenance and 2L EV treatment, appears to offer a promising strategy for long-term outcomes. Prospective studies are warranted to validate these findings and explore the implications of treatment sequencing and patient selection in the management of la/m UC.

## Figures and Tables

**Table 1:** Baseline demographics and clinical characteristics of patients in the 1L ddMVAC and GC subgroups at avelumab maintenance therapy initiation.

Characteristic, n (%)	1L PBC	
	ddMVAC (N = 32)	GC (N = 196)
**Age, median (range), years**	71 (49–82)	72 (43–87)
<65 years	10 (31.3)	44 (22.4)
≥65 years and <75 years	14 (43.8)	80 (40.8)
≥75 years and <80 years	5 (15.6)	49 (25.0)
≥80 years	3 (9.4)	23 (11.7)
**Sex**		
Male	23 (71.9)	147 (75.0)
Female	9 (28.1)	49 (25.0)
**BMI (kg/m^2^), median (IQR)**	23.1 (20.3–24.9)	23.0 (21.0–25.3)
<18.5 kg/m^2^	2 (6.3)	11 (5.6)
≥18.5 kg/m^2^ and <25 kg/m^2^	22 (68.8)	120 (61.2)
≥25 kg/m^2^	7 (21.9)	51 (26.0)
Unknown	1 (3.1)	14 (7.1)
**Smoking Status**		
No	10 (31.3)	51 (26.0)
Yes	21 (65.6)	142 (72.4)
Unknown	1 (3.1)	3 (1.5)
**ECOG PS**		
0	22 (68.8)	171 (87.2)
1	8 (25.0)	20 (10.2)
≥2	2 (6.3)	3 (1.5)
Unknown	0 (0.0)	2 (1.0)
**Primary tumor location**		
Ureter	7 (21.9)	34 (17.3)
Renal pelvis	8 (25.0)	50 (25.5)
Bladder	15 (46.9)	110 (56.1)
Urethra	2 (6.3)	2 (1.0)
**Metastatic site**		
Metastases	26 (81.3)	164 (83.7)
Regional lymph node	21 (65.6)	100 (51.0)
Distant lymph node	14 (43.8)	47 (24.0)
Visceral	15 (46.9)	60 (30.6)
Lung	8 (25.0)	40 (20.4)
Liver	4 (12.5)	11 (5.6)
Peritoneum	2 (6.3)	8 (4.1)
Other organs	2 (6.3)	6 (3.1)
Bone	3 (9.4)	33 (16.8)
Other	0 (0.0)	6 (3.1)
**Variant histology**		
Pure UC	24 (75.0)	147 (75.0)
UC with variant	2 (6.3)	32 (16.3)
Pure non-UC	2 (6.3)	2 (1.0)
Unknown	4 (12.5)	15 (7.7)
**Radical surgery history**		
No	21 (65.6)	115 (58.7)
Yes	11 (34.4)	81 (41.3)
**Adjuvant and neoadjuvant history**		
No	29 (90.6)	161 (82.1)
Yes	3 (9.4)	32 (16.3)
Unknown	0 (0.0)	3 (1.5)
**Hb (g/dL), median (IQR)**	9.6 (9.0–11.5)	10.7 (9.9–11.7)
<10 g/dL	18 (56.3)	52 (26.5)
≥10 g/dL	14 (43.8)	142 (72.4)
Unknown	0 (0.0)	2 (1.0)
**NLR, median (IQR)**	3.00 (2.17–4.31)	2.31 (1.58–3.27)
<3	15 (46.9)	132 (67.3)
≥3	16 (50.0)	61 (31.1)
Unknown	1 (3.1)	3 (1.5)
**CRP (mg/dL), median (IQR)**	0.10 (0.03–0.37)	0.15 (0.07–0.43)
≤0.3 mg/dL	22 (68.8)	131 (66.8)
>0.3 mg/dL	9 (28.1)	60 (30.6)
Unknown	1 (3.1)	5 (2.6)
**CCr (mL/min), median (IQR)**	65.0 (43.4–83.0)	57.2 (45.2–69.3)
≥60 mL/min	18 (56.3)	81 (41.3)
<60 mL/min	13 (40.6)	107 (54.6)
Unknown	1 (3.1)	8 (4.1)

1L, first-line; BMI, body mass index; CCr, creatinine clearance; CRP, c-reactive protein; ddMVAC, dose-dense methotrexate, vinblastine, doxorubicin, and cisplatin; ECOG PS, Eastern Cooperative Oncology Group Performance Status; GC, gemcitabine + cisplatin; Hb, hemoglobin; IQR, interquartile range; NLR, neutrophil lymphocyte ratio; PBC, platinum-based chemotherapy; UC, urothelial carcinoma.

**Table 2:** Characteristics of patients in the 1L ddMVAC and GC subgroups at 1L PBC initiation.

Characteristic, n (%)	1L PBC Regimen	
	ddMVAC (N = 32)	GC (N = 196)
**Platinum eligibility at 1L PBC initiation**		
Cisplatin eligible	16 (50.0)	78 (39.8)
Cisplatin ineligible ^a^/platinum eligible	14 (43.8)	91 (46.4)
Platinum ineligible ^b^	1 (3.1)	2 (1.0)
Unknown	1 (3.1)	25 (12.8)
**Number of cycles, median (IQR)**	4.0 (4.0–4.0)	4.0 (4.0–5.0)
1–3 cycles	4 (12.5)	37 (18.9)
4 cycles	22 (68.8)	109 (55.6)
5, 6 cycles	6 (18.8)	38 (19.4)
≥7 cycles	0 (0.0)	12 (6.1)
**Duration (weeks), median (IQR)**	13.2 (11.2–17.1)	21.1 (17.4–27.1)
**Platinum dose reduction, n (%)**	14 (43.8)	74 (37.8)
**First cycle when platinum dose reduction occurred, median (IQR)**	1.0 (1.0–2.0)	1.5 (1.0–2.0)
1 cycle	8 (25.0)	37 (18.9)
2 cycles	3 (9.4)	20 (10.2)
3 cycles	2 (6.3)	8 (4.1)
≥4 cycles	1 (3.1)	9 (4.6)
**TFI (weeks), median (IQR)**	4.2 (3.2–6.1)	5.1 (3.6–7.4)
<4 weeks	12 (37.5)	51 (26.0)
4 to 10 weeks	19 (59.4)	119 (60.7)
>10 weeks	1 (3.1)	26 (13.3)
**BOR**		
CR	3 (9.4)	21 (10.7)
PR	21 (65.6)	103 (52.6)
SD	8 (25.0)	72 (36.7)

^a^ According to this definition, cisplatin-unfit patients would meet at least one of the following criteria: ECOG performance status of 2, creatinine clearance < 60 mL/min, grade ≥ 2 hearing loss, grade ≥ 2 neuropathy, and/or New York Heart Association Class III heart failure [22]. ^b^ Platinum ineligibility was defined as ECOG performance status of ≤ 3 and creatinine clearance < 30 mL/min or ECOG performance status of 2 and creatinine clearance of < 45 mL/min [23]. 1L, first-line; BOR, best overall response; CR, complete response; ddMVAC, dose-dense methotrexate, vinblastine, doxorubicin, and cisplatin; GC, gemcitabine + cisplatin; IQR, interquartile range; PBC, platinum-based chemotherapy; PR, partial response; SD, stable disease; TFI, treatment-free interval.

**Table 3:** Summary of BOR with avelumab maintenance therapy.

	1L PBC	
	ddMVAC (N = 32)	Cisplatin/Gemcitabine (N = 196)
**Number of cases with response evaluation, *n***	26	173
**BOR, n (%)**		
CR	5 (19.2)	20 (10.2)
PR	5 (19.2)	23 (11.7)
SD	11 (42.3)	58 (29.6)
Non-CR/non-PD	0 (0.0)	9 (4.6)
PD	4 (15.4)	61 (31.1)
NE	1 (3.8)	2 (1.0)
**ORR%, (95% CI) ^a,^** **^c^**	38.5 (20.2–59.4)	24.9 (18.6–32.0)
**DCR% (95% CI) ^b,c^**	80.8 (60.6–93.4)	63.6 (55.9–70.8)

^a^ ORR (%) = (CR + PR)/(CR + PR + SD + Non-CR/non-PD + PD + NE) × 100. ^b^ DCR (%) = (CR + PR + SD + Non-CR/non-PD)/(CR + PR + SD + Non-CR/non-PD + PD + NE) × 100. ^c^ 95% CI is estimated using Clopper-Pearson method. BOR, best overall response, CI, confidence interval; CR, complete response; DCR, disease control rate; ddMVAC, dose-dense methotrexate, vinblastine, doxorubicin, and cisplatin; GC, gemcitabine + cisplatin; NE, not evaluable; ORR, objective response rate; PBC, platinum-based chemotherapy; PR, partial response; PD, progressive disease; SD, stable disease.

## Data Availability

Any requests for data by qualified scientific and medical researchers for legitimate research purposes will be subject to Merck’s Data Sharing Policy. All requests should be submitted in writing to Merck’s data sharing portal (https://www.merckgroup.com/en/research/our-approach-to-research-and-development/healthcare/clinical-trials/commitment-responsible-data-sharing.html [accessed on 28 March 2025]). When Merck has a co-research, co-development, or co-marketing or co-promotion agreement, or when the product has been out-licensed, the responsibility for disclosure might be dependent on the agreement between parties. Under these circumstances, Merck will endeavor to gain agreement to share data in response to requests.

## Supplementary Materials

The following supporting information can be downloaded at: https://www.mdpi.com/article/10.3390/curroncol32110618/s1, Table S1: Differences in characteristics and laboratory values between the start of 1L PBC and avelumab maintenance.
