# Supplementary material for: A Multicenter Retrospective Study of Avelumab First-Line Maintenance and Subsequent Therapies for Locally Advanced and Metastatic Urothelial Carcinoma: Subgroup Analysis of First-Line Dose-Dense Methotrexate, Vinblastine, Doxorubicin, and Cisplatin, and Gemcitabine Plus Cisplatin in the Japan AVElumab MAintenance and Continuous Treatment Study (JAVEMACS)
Source: Curr Oncol. 2025 Nov 5;32(11):618. doi: 10.3390/curroncol32110618 (PMC12651103; doi:10.3390/curroncol32110618)
Supplement: Supplementary file 1 [file curroncol-32-00618-s001.zip › curroncol-3890968-supplementary.pdf]

Supplementary

Supplementary Table S1. Differences in characteristics and laboratory values between the start of 1L PBC and avelumab maintenance.

Characteristic, n (%)	1L PBC Regimen			
	ddMVAC (*n* = 32)		GC (*n* = 196)	
	At the initiation of PBC	At the initiation of Avelumab	At the initiation of PBC	At the initiation of Avelumab
**BMI (kg/m^2), median (IQR)**	23.3 (21.0–25.1)	23.1 (20.3–24.9)	23.1 (21.1–25.3)	23.0 (21.0–25.3)
<18.5 kg/m^2	1 (3.1)	2 (6.3)	9 (4.6)	11 (5.6)
≥18.5 kg/m^2 and <25 kg/m^2	23 (71.9)	22 (68.8)	120 (61.2)	120 (61.2)
≥25 kg/m^2	8 (25.0)	7 (21.9)	49 (25.0)	51 (26.0)
Unknown	0 (0.0)	1 (3.1)	18 (9.2)	14 (7.1)
**ECOG PS**				
0	26 (81.3)	22 (68.8)	153 (78.1)	171 (87.2)
1	5 (15.6)	8 (25.0)	19 (9.7)	20 (10.2)
≥2	1 (3.1)	2 (6.3)	5 (2.6)	3 (1.5)
Unknown	0 (0.0)	0 (0.0)	19 (9.7)	2 (1.0)
**Hb (g/dL), median (IQR)**	12.3 (11.0–13.6)	9.6 (9.0–11.5)	12.3 (11.0–13.4)	10.7 (9.9–11.7)
<10 g/dL	1 (3.1)	18 (56.3)	25 (12.8)	52 (26.5)
≥10 g/dL	31 (96.9)	14 (43.8)	152 (77.6)	142 (72.4)
Unknown	0 (0.0)	0 (0.0)	19 (9.7)	2 (1.0)
**NLR, median (IQR)**	3.02 (1.85–6.39)	3.00 (2.17–4.31)	2.99 (2.02–4.71)	2.31 (1.58–3.27)
<3	15 (46.9)	15 (46.9)	87 (44.4)	132 (67.3)
≥3	15 (46.9)	16 (50.0)	84 (42.9)	61 (31.1)
Unknown	2 (6.3)	1 (3.1)	25 (12.8)	3 (1.5)
**CRP (mg/dL), median (IQR)**	0.32 (0.09–2.46)	0.10 (0.03–0.37)	0.57 (0.10–2.45)	0.15 (0.07–0.43)
≤0.3 mg/dL	16 (50.0)	22 (68.8)	73 (37.2)	131 (66.8)
>0.3 mg/dL	16 (50.0)	9 (28.1)	100 (51.0)	60 (30.6)
Unknown	0 (0.0)	1 (3.1)	23 (11.7)	5 (2.6)
**CCr (mL/min), median (IQR)**	67.2 (45.4–88.0)	65.0 (43.4–83.0)	59.6 (48.8–78.4)	57.2 (45.2–69.3)
≥60 mL/min	19 (59.4)	18 (56.3)	84 (42.9)	81 (41.3)
<60 mL/min	13 (40.6)	13 (40.6)	90 (45.9)	107 (54.6)
Unknown	0 (0.0)	1 (3.1)	22 (11.2)	8 (4.1)

1L, first-line; BMI, body mass index; CCr, Creatinine Clearance; CRP, c-reactive protein; ddMVAC, dose-dense methotrexate, vinblastine, doxorubicin, and cisplatin; ECOG PS, Eastern Cooperative Oncology Group Performance Status; GC, gemcitabine + cisplatin; Hb, hemoglobin; IQR, interquartile range; NLR, neutrophil lymphocyte ratio; PBC, platinum-based chemotherapy.
